# Clinical characteristics and factors affecting disease severity in hospitalized tick-borne encephalitis patients in Norway from 2018 to 2022

**DOI:** 10.1007/s10096-024-04855-2

**Published:** 2024-05-27

**Authors:** Hilde Skudal, Åslaug Rudjord Lorentzen, Tore Stenstad, Else Quist-Paulsen, Jens Egeland, Børre Fevang, Keson Jaioun, Bjørn Åsheim Hansen, Anne Marit Solheim, Yngvar Tveten, Malin Veje, Randi Eikeland, Hege Kersten

**Affiliations:** 1 Department of Infectious Diseases, Telemark Hospital Trust, Skien, Norway; 2 Institute of Clinical Medicine, Faculty of Medicine, Oslo University Hospital, Oslo, Norway; 3 Department of Neurology, Sørlandet Hospital Trust, Kristiansand, Norway; 4 Norwegian National Advisory Unit on Tick-borne Diseases, Sørlandet Hospital Trust, Kristiansand, Norway; 5 Department of Infectious Diseases, Vestfold Hospital Trust, Tønsberg, Norway; 6 Department of Microbiology, Oslo University Hospital, Oslo, Norway; 7 Division of Mental Health and Addiction, Vestfold Hospital Trust, Tønsberg, Norway; 8 Department of Psychology, University of Oslo, Oslo, Norway; 9 Section of Clinical Immunology and Infectious Diseases, Department of Rheumatology Dermatology and Infectious Diseases, Oslo University Hospital, Oslo, Norway; 10 Department of Research, Telemark Hospital Trust, Skien, Norway; 11 Institute of Clinical Medicine, University of Bergen, Bergen, Norway; 12 Department of Clinical Microbiology, Telemark Hospital Trust, Skien, Norway; 13 Department of Infectious Diseases, Region Västra Götaland, Sahlgrenska University Hospital, Gothenburg, Sweden; 14 Institute of Biomedicine, Department of Infectious Diseases, University of Gothenburg, Gothenburg, Sweden; 15 Faculty of Health and Sport Sciences, University of Agder, Grimstad, Norway

**Keywords:** Tick-borne encephalitis, TBE, Clinical characteristics, Meningitis, Encephalitis, Myelitis

## Abstract

**Purpose:**

To describe the clinical characteristics and factors associated with disease severity in a Norwegian cohort of hospitalized patients with tick-borne encephalitis (TBE).

**Methods:**

This observational multicenter study included hospitalized patients with TBE in the endemic area in the southeastern region of Norway from 2018 to 2022. Clinical signs and findings from laboratory tests, EEG, CT and MRI scans were recorded. Patient characteristics were compared among those with mild, moderate, and severe TBE, and factors associated with disease severity were identified.

**Results:**

Nearly all eligible patients were included in the final cohort (153/189 participants, 81%). The median age was 56 years, 63% were men, and 7% were vaccinated against TBE; no participants were fully vaccinated. TBE presented as mild (meningeal) disease in 31% of patients and as moderate or severe (encephalitic) disease in 54% and 14% of patients, respectively. We found that 46% of the patients had a monophasic course, 64% had hyponatremia, and 7% presented with central nervous system (CNS) symptoms without pleocytosis in cerebrospinal fluid (CSF). Dysesthesia, a symptom previously not described, was reported in 10% of the patients. Most objective findings were related to the CNS. Preexisting comorbidities, CRP and CSF protein levels were predictors of more severe disease.

**Conclusion:**

This novel presentation of a large Norwegian cohort supports TBE as a serious disease in the southeastern region of Norway. The majority of hospitalized patients presented with encephalitis, and fewer presented with meningitis. Comorbidities, CRP and CSF protein levels were associated with more severe disease.

**Trial registration:**

Prosjekt #2,296,959 – The Norwegian Tick-borne Encephalitis Study – NOTES. Acute phase characteristics and long-term outcomes. – Cristin.

**Supplementary Information:**

The online version contains supplementary material available at 10.1007/s10096-024-04855-2.

## Introduction

Tick-borne encephalitis (TBE) is an infection of the central nervous system (CNS) caused by the TBE virus (TBEV). TBE represents a serious health risk in parts of Europe [1], the Russian Federation and northern Asia [2].

Mainly three different subtypes of TBEV have been described to cause human disease, with different geographic distribution and severity [3]; the European subtype (TBEV-Eu) is transmitted mainly by *Ixodes ricinus*, while the Far Eastern (TBEV-FE) and Siberian (TBEV-Sib) subtypes are transmitted mainly by *I. persulcatus.* In Norway, only TBEV-Eu has been detected thus far [4]. Infections with TBEV are often asymptomatic, and the clinical presentation of TBE varies from mild febrile disease to meningitis, encephalitis or meningoencephalomyelitis [2, 5]. The course of TBE is reported to be biphasic in 65–75% of cases, in which an initial viremic phase is followed by an asymptomatic interval lasting approximately one week (1–33 days) before a second phase involving CNS infection [6–8]. In contrast, a monophasic course has no initial phase followed by asymptomatic interval preceding the onset of CNS symptoms [9].

The fatality rate in Europe is less than 1% [1]. Nevertheless, long-term neurological sequelae are common and have been reported in up to 40–50% of patients in European studies [10, 11]. No specific antiviral therapy is available, but TBE is highly preventable by vaccination [5]. Nonetheless, vaccination rates are low in many European countries, probably due to lack of awareness, demanding vaccination regime consisting of multiple dosages, and high vaccination costs [12].

Since 2015, the incidence of TBE in Europe has increased markedly [1, 13], leading to increased morbidity and health care costs [14]. Climate changes, with enlarged endemic areas, extension of the season for transmission and possibly changes in leisure habits, are proposed contributing factors [15].

In Norway, TBE has been a mandatory notifiable disease since 1975, but the disease was not diagnosed in Norway until 1997 [16]. The incidence of TBE has been low, with an average of less than seven reported cases every year from 1997 to 2017. Since 2018, the number of cases has increased markedly to more than 80 in 2022. Surveillance studies have shown that the majority of the cases (99%) are associated with tick bites in the coastal areas in the southern part of Norway in Agder, Vestfold and Telemark counties [17].

The burden of TBE in Norway is unknown, as only case reports of TBE have been published [16, 18]. Hence, the course and severity of Norwegian TBE infections have not been previously described, and an understanding of the clinical and immunological features and long-term outcomes is needed.

Herein, we present clinical symptoms, objective signs, and results from investigations, such as laboratory tests, magnetic resonance imaging (MRI), computerized tomography (CT) scans and electroencephalography (EEG), in an almost comprehensive Norwegian cohort of hospitalized TBE patients. We further aim to identify factors predictive of a severe disease course.

## Materials and methods

### Participants

In this observational multicenter study, we recruited hospitalized patients with confirmed TBE in the endemic area of Norway from 2018 to 2022. All the included patients sought medical attention and were admitted to one of the three hospitals providing inpatient acute care in the region: Telemark, Vestfold or Sørlandet Hospital.

The inclusion criteria were as follows: (i) confirmed TBE (ECDC case definition) [19], with symptoms or signs of inflammation in the CNS (e.g., meningitis, meningoencephalitis, or meningoencephalomyelitis) and at least one of the following: TBEV-IgM and TBEV-IgG antibodies in the blood and/or TBEV-IgM in the cerebrospinal fluid (CSF) and/or seroconversion of TBEV-IgG in paired serum samples or detection of TBEV RNA by real-time polymerase chain reaction (PCR) in clinical specimens; (ii) aged ≥ 16 years; (iii) verified or suspected exposure to ticks in southeastern Norway; and (iv) hospitalized in participating hospitals. Patients vaccinated for TBE were included if infection with TBEV was confirmed by intrathecal production of either TBEV-IgG or TBEV-IgM in CSF or ≥ 10 months since the last vaccination dose was given when clinical and laboratory findings supported the diagnosis (CSF pleocytosis > 5 × 10^6^ cells/L and both TBEV-IgM and TBEV-IgG in serum) [20].

Patients with demonstrated intrathecal synthesis of borrelia-specific antibodies and CSF pleocytosis > 5 × 10^6^ cells/L plus confirmed TBE were considered to have possible concomitant infections with TBEV and *Borrelia burgdorferi sensu lato*.

### Data collection

TBE patients were identified prospectively during the hospital stay, or shortly after discharge, or retrospectively through identification after a review of microbiological files or at the routine follow-up visit. Retrospective data were obtained by retrieving data from the participants’ medical records and/or by interview and clinical investigation at follow-ups. In cases of missing data in medical records, the participants were contacted by phone to complete the dataset.

For all patients, data on sociodemographics, comorbidities, medications, known tick bites, TBE vaccination status, date of admission, length of hospital stay and results from investigations were recorded. Immunodeficiency was defined as active cancer, both hematologic malignancy and solid tumors, other severe chronic disease or immunosuppressive conditions, including primary immunodeficiencies, HIV infection, organ transplantation and cytostatic or other immunosuppressive treatment. The duration of the acute phase was defined as the number of days from the onset of symptoms in the CNS phase until discharge, but the number of “days from symptom onset to admission” was counted from the first day of symptoms in the first phase. A modified clinical composite score (CCS) comprising 39 items (supplement 1) was designed and recorded for all patients. The CCS was originally developed for the assessment of Lyme neuroborreliosis [21], and in this modified version, additional variables previously used for the assessment of TBE were included [22]. The modified CCS measures 17 symptoms based on information from the anamnesis according to a standardized list, and 22 objective neurological findings detected through clinical examination of both the peripheral nervous system (PNS) and CNS. The 39 items were scored as 0 = none, 1 = mild (without influence on daily life) or 2 = severe (with influence on daily life). Thus, the sum of the scores ranged from 0 to 78. The sum of scores for subjective symptoms; objective findings from the PNS and CNS; and the total score were recorded. Other relevant symptoms, beyond those we inquired about in the CCS, were recorded if reported by the investigator.

### Classification of disease severity

Disease severity was classified as mild, moderate or severe in accordance with previously published classifications [22, 23].

Mild disease was defined as meningeal symptoms with fever, headache, nausea, vomiting, neck stiffness, and sensitivity to light and/or sound with either normal or not-performed EEG. Moderate disease was defined as slightly altered consciousness and/or diffuse neurological symptoms such as confusion, slow thinking, or focal neurological symptoms such as ataxia, tremor and dysphasia. Severe disease was defined as multifocal symptoms and/or severe signs of encephalitis with altered consciousness.

Cognitive symptoms assessed by clinical observation were subgrouped into mental slowness, altered consciousness and confusion. Moreover, we assessed clinically whether the patients had symptoms and/or findings consistent with myelitis.

### Microbiology, biobanking and supplementary investigations

Blood samples were collected from all patients. CSF and whole-blood samples were collected for biobanking from the patients included prospectively. TBEV-IgM and IgG were analyzed using the ELISA TICK-BORNE ENCEPHALITIS VIRCLIA® IgM/IgG MONOTEST (Vircell, Granada, Spain) or Serion Elisa classic TBE-virus IgG/IgM (Serion diagnostics, Würzburg, Germany). During the study period, a rapid IgM test, which detects IgM reactive to TBEV proteins (ReaScan TBE IgM rapid test [Reagena Oy Ltd., Toivala, Finland]) was implemented and analyzed for patients in whom TBE was suspected. Positive results were confirmed with an ELISA-test. In-depth analyses of serum and/or CSF samples were performed with PCR to detect TBEV RNA if antibody tests were negative, and if TBE remained clinically suspected.

The clinicians at each site considered the indications for EEG, MRI and CT scans of the brain.

### Statistical analyses

The normality of continuous variables was assessed by the Shapiro‒Wilk test. Normally distributed variables are summarized using mean and standard deviation (SD), while median and interquartile range (IQR) are presented for non-normally distributed variables. The variables in Tables 1, 2 and 3 are summarized and categorized according to the TBE severity group: mild, moderate and severe. Patient characteristics were compared between the TBE severity groups using the Kruskal‒Wallis test or one-way ANOVA, as appropriate. For multiple comparisons, Dunn’s test or Tukey’s test was applied with Bonferroni correction, and the presented p values were “Bonferroni adjusted”, where the familywise error rate was adjusted by multiplying the p value in each pairwise test by the number of comparisons. Pearson’s chi-square test or Fisher’s exact test was applied to compare categorical variables. Correlations between variables were estimated using Pearson’s or Spearman’s rank correlation coefficient ($\rho$*)*, as appropriate. Univariate and multivariate multinomial logistic regression analyses were used to identify the risk factors for disease severity. Independent variables with a p value < 0.2 in the univariate models were assessed for multicollinearity (variance inflation factor < 5) before entered into the multivariate model. Analyses were performed in Stata version 17.0 (StataCorp, College Station, TX, USA). Unadjusted and adjusted P values < 0.05 were considered to indicate statistical significance.

**Table 1:** Patient characteristics and findings stratified by disease severity

Characteristics		All patients *n* = 153	Severity			*p* value^‡^
			Mild *n* = 48	Moderate *n* = 83	Severe *n* = 22	
Age at admission, median (IQR)		56 (43–66)	52 (39–65)	56 (45–68)	60 (48–68)	0.297
Sex, *n* (%)	Male	96 (62.7)	37 (77.0)	46 (55.4)	13 (59.1)	0.043^*^
Any comorbidity, *n* (%)		83 (54.3)	26 (54.2)	39 (47.0)	18 (81.8)	0.014^*^
Immunosuppression, *n* (%)		9 (5.9)	4 (8.3)	3 (3.6)	2 (9.1)	0.311
Previous TBE vaccination, *n* (%)	Yes	11 (7.3)	4 (8.5)	5 (6.0)	2 (9.5)	0.744
History of tick bite, *n* (%)	Yes	128 (83.7)	40 (83.3)	70 (84.3)	18 (81.8)	0.954
Biphasic course, *n* (%)	Yes	81 (53.3)	26 (54.2)	46 (56.1)	9 (40.9)	0.443
Fever, *n %*		141 (92.1)	43 (89.6)	76 (91.6)	22 (100)	0.308
Days between symptom onset and admission, median (IQR)		14 (7–19)	14 (8–20)	14 (8–20)	8.5 (4–14)	0.004^*†^
Length of stay (day), median (IQR)		6 (4–9)	4 (3–7)	6 (4–9)	10 (8–35)	< 0.001^*†^
**Clinical Composite Score**						
-Total Clinical Composite Score, mean (SD)		18.1 (7.8)	12.3 (5.9)	20.0 (6.5)	23.2 (8.7)	< 0.001^*†^
-Symptoms Score,, mean (SD)		13.5 (5.1)	11.6 (5.5)	14.6 (4.6)	13.8 (4.8)	0.004^*†^
-Objective Sum Score, CNS, median (IQR)		2 (1–7)	0 (0–1)	4 (2–7)	8 (5–12)	< 0.001^*†^
-Objective Sum Score, PNS, median (IQR)		0 (0–1)	0 (0–0)	0 (0–1)	0 (0–2)	< 0.001^*†^

IQR, interquartile range; SD, standard deviation; CNS, central nervous system; PNS, peripheral nervous system; TBE, tick-borne encephalitis

‡ Pearson’s chi-square test, Fisher’s exact test, one-way ANOVA or the Kruskal‒Wallis test were used as appropriate

† Details significant for comparison test with Bonferroni adjustment: Days between symptom onset and admission, mild vs. severe (adj. p value = 0.005), moderate vs. severe (adj. p value = 0.002); length of stay, mild vs. moderate (adj. p value = 0.013), mild vs. severe (adj. p value < 0.001), moderate vs. severe (adj. p value < 0.001); Total Clinical Composite Score, mild vs. moderate (adj. p value < 0.001), mild vs. severe (adj. p value < 0.001); Symptoms Score, mild vs. moderate (adj. p value = 0.009); Objective Sum Score, CNS, mild vs. moderate (adj. p value < 0.001), mild vs. severe (adj. p value < 0.001), moderate vs. severe (adj. p value = 0.007);Objective Sum Score, PNS, mild vs. moderate (adj. p value = 0.005), mild vs. severe (adj. p value < 0.001)

* p value < 0.05

**Table 2:** Laboratory, EEG and radiological findings stratified by disease severity

Laboratory findings		All patients *n* = 153	Severity			*p* value^‡^
			Mild *n* = 48	Moderate *n* = 83	Severe *n* = 22	
**Blood**						
Leukocytes (at admission) × 10 ^9^/L, median (IQR), *n* = 152		10.1 (7.3–12.1)	9.7 (6.9–11.7)	10.1 (7.4–12.0)	11.5 (7.4–13.0)	0.420
CRP (highest value registered during stay) mg/L, median (IQR)		19.0 (8.0–41.0)	12.0 (5.0-21.5)	18.0 (8.0–41.0)	30.0 (26.0–66.0)	< 0.001^*†^
Hyponatremia ^ξ^,	n (%)	97 (63.4)	25 (52.1)	56 (67.5)	16 (72.7)	0.131
	(Lowest value registered during stay) mmol/L, median (IQR), *n* = 97	133 (130–134)	133 (130–134)	134 (132–136)	129 (128–132)	< 0.001^*†^
**CSF**						
Lumbar puncture, n (%)		144 (94.1)	42 (87.5)	80 (96.4)	22 (100.0)	0.051
Pleocytosis (> 5 leukocytes, x 10 ^9^/L), n (%) *n* = 143^a^		132 (92.3)	36 (85.7)	75 (94.9)	21 (95.5)	0.164
Leukocytes x 10 ^9^/L, median (IQR), *n* = 132^b^		71 (35–130)	66 (28–122)	67 (36–131)	96 (53–150)	0.182
% monocytes, median (IQR)		77 (48–91)	78 (2–39)	77 (54–91)	69 (28.5–88.5)	0.655
Glucose ratio, median (IQR), *n* = 126		0.6 (0.5–0.6)	0.6 (0.5–0.6)	0.6 (0.5–0.6)	0.6 (0.5–0.7)	0.340
Protein, g/L, median (IQR), *n* = 144		0.69 (0.54–0.93)	0.59 (0.48–0.77)	0.71 (0.57–0.92)	0.83 (0.62–0.97)	0.025^*^
Albumin ratio, median (IQR), *n* = 97		12.3 (9.1–17.0)	9.4 (8.0–13.0)	12.6 (10.0-17.8)	16.3 (14.0-21.6)	0.005^*^
**EEG and radiological findings**						
EEG suggesting encephalitis (performed *n* = 59), n %		46/59 (78.0)	0/6 (0)	31/38 (81.6)	15/15 (100)	< 0.001^*^
CT caput suggesting encephalitis (performed *n* = 110), n %		3/110 (2.7)	0/32 (0)	2/58 (3.5)	1/20 (5.0)	-
MRI cerebri suggesting encephalitis (performed *n* = 96), n %		17/96 (17.7)	0/18 (0)	11/60 (18.3)	6/18 (33.3)	0.018^*^

The median blood neutrophil, lymphocyte, and platelet counts were within the normal range (not shown in Table 2), with the exception of the white blood cell count, which was slightly above the normal range. We identified 22 (18.2%) patients (8 with mild disease, 9 with moderate disease and 5 with severe disease) with increased transaminase levels. No significant differences in blood test results between the study groups were observed

IQR, interquartile range; TBE, tick-borne encephalitis; CSF, cerebrospinal fluid; S, serum; EEG, electroencephalography; CT, computerized tomography; MRI, magnetic resonance imaging

‡ Kruskal–Wallis test, Fisher’s exact test or Pearson’s chi-square test were used as appropriate

† Details significant for comparison test with Bonferroni adjustment: CRP, mild vs. severe (adj. p value < 0.001), moderate vs. severe (adj. p value = 0.012); hyponatremia, mild vs. moderate (adj. p value = 0.030), mild vs. severe (adj. p value = 0.033), moderate vs. severe (adj. p value < 0.001); Sp-protein, mild vs. severe (adj. p value = 0.013); Albumin ratio, milld vs. moderate (adj. p value = 0.044); mild vs. severe (adj. p value = 0.002)

ξ Hyponatremia: only values less than the normal range were recorded; not applicable

* p value < 0.05

^a^143/144 CSF samples were analyzed for leukocytes

^b^Leukocytes in CSF are shown for CSF samples with pleocytosis

A ^c^A value < 10.2 was considered consistent with an intact blood‒brain barrier. Albumin ratio = CSF-albumin: Serum-albumin

**Table 3:** Multinomial logistic regression analysis of patient characteristics with disease severity as the dependent variable

Characteristic	Severity					
	Mild^a^ vs. Moderate		Mild^a^ vs. Severe		Moderate^a^ vs. Severe	
	RR (95% CI)	*p* value	RR (95% CI)	*p* value	RR (95% CI)	*p* value
Age	1.010 (0.982–1.039)	0.482	0.989 (0.949–1.032)	0.618	0.980 (0.942–1.018)	0.293
Sex, female†	4.576 (1.718–12.183)	0.002*	4.674 (1.187–18.402)	0.027*	1.021 (0.306–3.408)	0.972
Monophasic course	0.878 (0.341–2.264)	0.788	0.485 (0.116–2.023)	0.321	0.552 (0.151–2.019)	0.369
Hyponatremia, yes‡	1.887 (0.776–4.588)	0.161	2.563 (0.660–9.957)	0.174	1.358 (0.376–4.902)	0.640
Comorbidity, yes‡	0.431 (0.173–1.071)	0.070	3.552 (0.761–16.170)	0.105	8.181 (1.915–34.944)	0.005*
Debut of symptoms to admission	1.005 (0.937–1.078)	0.883	0.889 (0.794–0.996)	0.043,*	0.885 (0.797–0.982)	0.021*
CRP	1.000 (0.986–1.014)	0.990	1.016 (1.002–1.030)	0.028*	1.016 (1.002–1.029)	0.026*
CSF-protein	9.243 (1.737–49.180)	0.009*	10.662 (1.206–93.574)	0.033*	1.149 (0.187–7.051)	0.881

† the reference group is male; ‡ the reference group is no; ^a^ the reference outcome; * significant level p value < 0.05, RR, relative risk ratio; CI, confidence interval; CSF, cerebrospinal fluid

### Ethical considerations

The study was approved by the South-Eastern Regional Ethical Committee (Study no 96505) and the Data Protection Office at all the participating centers. Written informed consent was obtained from all the participants.

## Results

**Fig. 1:**
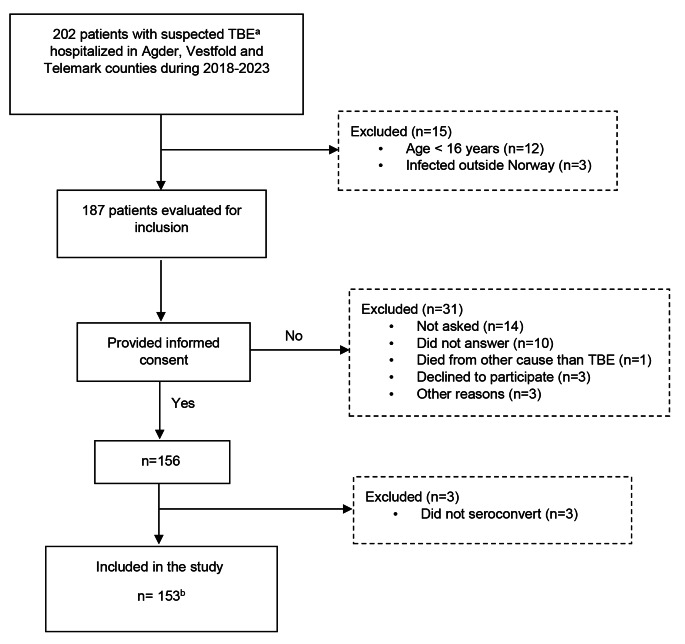
Patient enrolment. ^a^ Confirmed TBE or clinically suspected TBE with or without positive TBEV-IgM. ^b^ Seventy-seven patients were included during hospitalization and 12 shortly after discharge. Data were obtained from the medical records of 31 patients. Moreover, 33 patients were included at the three- (*n* = 3), 12- (*n* = 22) and > 18 (*n* = 8)-month follow-up visits after TBE hospitalization

A total of 153 TBE patients were included (Fig. 1). All patients were admitted between April and December, with peaks in July (*n* = 40), August (*n* = 31) and September (*n* = 31). The age span was 17–87 years (median 56 years), 63% were men, and more than half of the patients were classified as having moderate TBE disease (Table 1). One or more comorbidities were reported by 54% of the patients, and those with severe TBE had more comorbidities than did the mild and moderately affected patients. The prevalence of any single illness did not differ between the TBE severity groups (data not shown). The most frequent comorbidities identified were hypertension (20%), allergies (14%) and chronic heart disease (11%). Although 11 participants had received previous vaccination dosage(s), no patient was fully vaccinated. Among the nine patients classified as immunocompromised, two had severe disease, and three were incompletely vaccinated against TBE.

46% of the patients had a monophasic course. The patients with a monophasic course were older (*p* = 0.035) compared to those with a biphasic course. No correlation was found between the monophasic course and disease severity or comorbidity (chi-square = 1.629, significance level p value = 0.443 and chi-square = 2.917, p value = 0.088, respectively).

The median time from symptom onset to admission was 14 days (range 1–50), and the median length of hospital stay was six days (range 0-109). Patients with severe disease had a significantly shorter time to admission and longer hospital stay than patients with both mild and moderate disease. Of the 22 patients classified as severely ill, eight were admitted to the intensive care unit (ICU), and four had seizures during the hospital stay. The only TBE-related death occurred in a previously active 78-year-old male with hypertension, who died 11 weeks after TBE onset.

### Symptoms

All but two patients reported malaise, while 92% reported fatigue. Other frequently reported symptoms were headache (85%), balance disturbance (74%) and nausea or vomiting (73%). Approximately two-thirds of the participants experienced cognitive symptoms, namely, impaired attention (65%) and memory difficulties (41%). Insomnia was reported by 41% of patients.

Dysesthesia, which was not intentionally inquired about through a specific question, was reported by 10% of patients, either at admission or just before deterioration prior to admission. The sensation was described by the patients as a sunburn-like feeling, disappearing after one or a few days, and was located symmetrically on either lateral side of the legs, on the scalp, on the back, or even as a painful belt around the trunk. Due to this complaint, some patients saw a doctor and even received analgesics, but none were treated with antibiotics.

### Objective findings

**Fig. 2:**
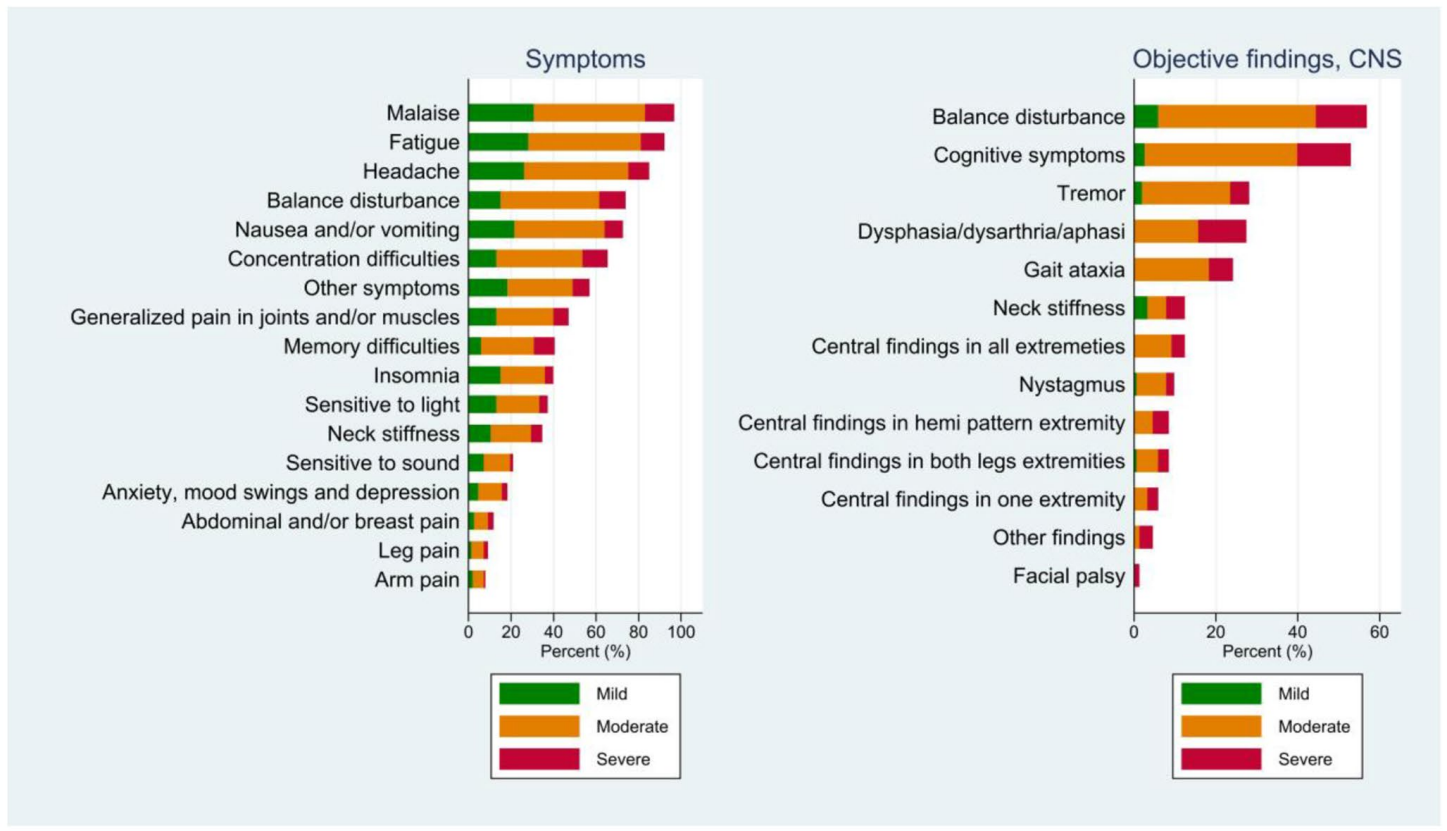
Symptoms and findings stratified by TBE disease severity in the CNS phase. Each bar represents the total percentage of symptoms or objective findings. The colors show the percentages for each severity group: mild, moderate and severe. The following symptoms were recorded as “other symptoms”: diarrhoea *n* = 27 (18%); dizziness, *n* = 23 (15%); reduced appetite, *n* = 23 (15%); diplopia, *n* = 11 (7%); weight loss > 5 kg, *n* = 16 (11%); and dysesthesia, *n* = 15 10%

The majority of the objective findings were related to the involvement of the CNS (Fig. 2). Cognitive symptoms were observed in more than half (53%) of the patients, 95% of whom were classified as having moderate or severe disease. Despite no signs of cognitive symptoms during hospitalization, two patients were classified as having severe disease due to multifocal symptoms. The subclassification of cognitive symptoms showed slowness (48%), altered consciousness (30%) and/or confusion (29%). Almost 30% of the patients had findings in the extremities indicative of CNS involvement, including a positive plantar reflex, hyperreflexia, spasticity/hypertonia, slower mental speed or impaired coordination. Among these patients, all four limbs were affected in 12%. Tremor was observed in 28% of the patients. Facial palsy was recognized in two patients, both of central origin and with severe disease. Overall, there were few findings from the PNS, the most common being eye muscle paresis (8%) and nonradicular paresis (6%).

A diagnosis of myelitis was made for eight patients, and myelitis was strongly suspected due to respiratory failure and urinary retention in another five patients (8%). We found that patients with increasing disease severity, as defined by Veje et al. [22], had a significantly higher mean total CCS (data not shown).

### Laboratory tests and supplementary findings

#### Laboratory tests

Hyponatremia was found in 63% of patients. The patients with hyponatremia were older (*p* = 0.010), more frequently had severe TBE, had longer hospital stays (*p* < 0.002), and had a higher total CCS (*p* = 0.017) than the patients without hyponatremia. The level of CRP was moderately increased in the group with severe disease compared to the group with moderate or mild disease (Table 2). CSF was analyzed in 94% of the patients. The median levels of CSF-protein and the CSF/S albumin ratio increased with increasing disease severity.

Eleven patients out of 144 sampled patients had no CSF pleocytosis. Six of these non-pleocytic patients had mild TBE, four had moderate TBE, and one severe TBE. All non-pleocytic patients had a serologically verified diagnosis (IgM + IgG). Among them, two patients, one with mild- and the other with severe TBE, also had positive PCR with the detection of TBEV RNA in serum and CSF, respectively. Three patients had pathological EEG suggestive of encephalitis and five had isolated increased CSF-protein. Seven out of 11 patients reported known tick bite within the four weeks before admission. The ages ranged from 40 to 83 years, and all were unvaccinated. Six of the patients had comorbidities; chronic heart disease, diabetes mellitus, hypertension, rheumatic disease, and fibromyalgia/chronic fatigue-syndrome. One patient had lymphoma which was not in need of any treatment. Moreover, patients without pleocytosis were more likely to have a monophasic course (chi-square = 5.851, *p* = 0.016; monophasic 13.4% vs. biphasic 2.6%) and had significantly fewer days from symptom onset to admission than patients with pleocytosis (median 6 vs. 14 days, respectively; *p* = 0.003).

Lumbar puncture was performed in 144 of the patients. Culture and PCR analyses of other common viral causes of CNS infection were performed for 82% and 69% respectivly. All were negative.

The Borrelia IgG CSF/S ratio was determined for 85% (123/144) of the patients who underwent lumbar puncture, and six patients fulfilled the criteria for Lyme neuroborreliosis [24]. Of these, one had mild TBE, three had moderate TBE, and two had severe TBE (data not shown). For the patients with dysesthesia, the Borrelia IgG CSF/S ratio were analyzed and negative for 13 out of 15 patients. The last two patients had not detectable borrelia-specific antibodies in neither serum nor CSF.

Six patients, prospectively included, tested negative for TBE-IgM at initial testing. Three had a history of TBE vaccination, two of whom were classified as immunosuppressed. One patient was unvaccinated and immunosuppressed and was the only patient diagnosed through the detection of TBEV RNA (in CSF) by PCR after repeated tests were negative for TBE-IgM and TBE was still strongly suspected. Another patient was considered to be in the early CNS phase of TBE five days after the onset of symptoms until admission, while a male in his 70s was admitted 10 days after symptom onset. Five of the six patients had a monophasic course. TBE was subsequently confirmed in all patients according to the case definition.

### EEG, CT and MRI

The majority of patients in whom EEG was performed had findings suggestive of encephalitis (78%, 46/59), including all patients classified with severe TBE. Moreover, MRI were pathological in 18% (17/96), whereof 16% (15/96) indicated encephalitis, and 2% (2/96) had meningitis with contrast enhancement. Among the patients with abnormal findings on MRI, 11 patients had moderate disease, and six had severe disease. Three percentages (3/110) of the CT scans suggested encephalitis, all of which corresponded with the MRI findings.

### Predictors of TBE disease severity

According to the results of the multinomial logistic regression analysis (Table 3), the following factors were associated with more severe TBE disease: compared to men, women had an increased risk of having moderate or severe disease versus mild; patients with “any comorbidities” had an eightfold greater risk than patients without comorbidities of having severe versus moderate disease; and the number of days from symptom onset to admission were fewer for patients with severe disease. Moreover, having higher levels of CRP increased the risk of having severe disease compared to moderate and mild disease. Finally, having higher CSF protein levels increased the risk of having moderate or severe disease compared with mild disease.

## Discussion

This is the first description of a cohort consisting of the majority of TBE patients hospitalized in Norway over five years. During the study period, 259 TBE patients were reported to the Norwegian Surveillance System for Communicable Diseases (MSIS) [17]. Of the reported TBE patients, 93% required hospitalization, which is in accordance with a previous reported European study [3]. Of the patients reported to be infected in Norway, 189 were older than 16 years (unpublished data, MSIS, 2024); thus, we present a nearly complete cohort (153/189, 81%) with an in-depth description of the acute phase of TBE in patients in Norway.

The characteristics of the Norwegian TBE cohort were comparable to those of previously described TBE cohorts [7, 22]. Almost all patients had a high degree of malaise and symptoms related to the CNS, while only a limited number of the patients had PNS involvement. Few patients were diagnosed with myelitis (9%), which is somewhat lower than that reported in one large European multicenter study (13%) [7]. Neck stiffness was clinically demonstrated in almost 13% of the patients, which aligns with a previous Scandinavian study that reported the same finding in 17% of patients [22]. The overall number, however, increased to 35% when including those who reported symptomatic neck stiffness. This distinction might partially explain why Kohlmaier et al. reported a neck stiffness rate as high as 74% [7]. Tremor was recognized in 28% of the patients. Kohlmaier et al. reported a somewhat higher rate of 38% [7], while two retrospective studies reported rates of 16% and 32%, respectively [22, 25]. Noteworthy, in the present data, differences were observed between study centers, which may be attributable to inter-observer reporting variations.

Almost three-quarters of the patients were classified as having moderate or severe disease, which is roughly consistent with previous findings from Sweden and Lithuania using the same classification of disease severity [22, 23]. In a clinical context, moderate TBE disease is also considered a serious infection requiring hospitalization.

To the best of our knowledge, dysesthesia as a symptom of TBE has not been described previously. The described sensation is typically a girdle-like sensation around the trunk possibly indicating sensory myelitis or possible PNS-related radiculitis. On the other hand, other patients experienced symmetric dysesthesia of the scalp or the lateral aspects of the lower extremities. These sensations are incompatible with myelitis and the pathogenesis remains unkown. We suggest that this symptom may be an early sign of TBE and that the symptom might not be restricted to only the CNS phase; furthermore, coinfection with *Borrelia burgdorferi* could not explain this symptom in this patient cohort.

The biphasic course is well described as the typical course of European TBE in approximately 70% of patients [23, 25, 26]. However, recently, Bogovic et al. reported that 40% of patients had a monophasic course; as these patients were older, more likely to have comorbidities, more frequently admitted to the intensive care unit, and were more likely to have severe disease [27]. Similarly, in our cohort, we found that 46% of the patients had a monophasic course, confirming that a monophasic course is not an atypical presentation of TBE in Europe. We also found that a monophasic course and severe disease correlated with advanced age and that a greater proportion of severely affected patients had a monophasic course than did those with mild/moderate disease, which is in accordance with Bogovic et al. [27]. Moreover, the majority of the patients with a history of TBE vaccination (eight out of 11) and immunosuppression (seven out of nine) had a monophasic course.

Hyponatremia was observed in 63% of patients, while previous studies have reported hyponatremia in 32% and 44%, respectively [25, 28]. The patients with hyponatremia were older, had longer hospital stays and had more CNS-affecting findings than patients with normal blood sodium levels. Dehydration is the most frequent cause of hyponatremia in TBE patients [28], likely because of a high body temperature frequently exceeding 40 °C in the CNS phase of TBE [29], vomiting and poor fluid intake.

Our finding of 11 (7%) patients presenting with neuroinfection, including 5 patients with serious CNS infection but no pleocytosis in CSF, is novel, as only two cases have previously been convincingly reported [30, 31]. In the present study, we used the ECDC case definition [1], which intended to define TBE cases for public health surveillance. However, CSF pleocytosis is not included in this definition. In contrast, pleocytosis in CSF has been a diagnostic criterion for TBE in larger clinical studies [2, 23, 27, 32], as well as a criterion for aseptic meningitis and a minor criterion for encephalitis in general [33, 34]. Nevertheless, this phenomenon has been described in patients with encephalitis associated with herpes simplex virus and in aseptic meningitis [35–38]. For TBE, Bogovic et al. highlighted in a review from 2015 that TBE without CSF pleocytosis was confirmed by referring to one patient diagnosed with serious TBE without CSF pleocytosis [30]. Additionally, Gunther et al. have suggested that the finding of pleocytosis in TBE patients in previous clinical studies could be due to selection bias [39]. The non-pleocytic patients in the current study had a confirmed diagnosis of TBE through serological testing, of whom two also tested positive for TBEV RNA by PCR. Nevertheless, more recent clinical cohort studies which also has used the ECDC case definition [1], have to the best of our knowledge, not reported these findings of TBE patients without CSF pleocytosis [7, 22, 40]. In our study, the patients without pleocytosis had significantly fewer days from symptom onset to admission than did the patients with pleocytosis; therefore, the patients without pleocytosis were probably in an earlier stage of TBE than those with pleocytosis, and the immune response in the CNS may not have fully developed at the time of lumbar puncture. Notably, all 11 patients without pleocytosis were unvaccinated, and a key feature of vaccination is the rapid mobilization of the adaptive immune response. Repuncture later during the course of treatment could potentially have revealed pleocytosis in these patients. Nevertheless, our study suggests that CSF without evidence of pleocytosis can be observed not only during the course of mild disease but also in severe forms of TBE. Further investigations involving repeated lumbar punctures could provide additional information about the immune response, and these patients should be included in future studies.

Intrathecally produced borrelia antibodies were found in six of the patients. A positive borrelia antibody CSF/S ratio might indicate persistent intrathecally produced antibodies from previous Lyme neuroborreliosis or current coinfection [41].

Less than 3% of the CT scans performed suggested encephalitis, all with comparable findings on MRI. Almost 18% of the MRIs performed suggested encephalitis. Thus, the indication for CT scan might be limited to situations when immediate imaging is required or if MRI is contraindicated.

Like in previous studies, preexisting comorbidities were identified as risk factors for severe disease [7, 32]. Interestingly, patients with any comorbidity had an eightfold greater risk of having severe disease than moderate disease, although more than one-third of the reported comorbidities were well-treated asymptomatic diseases. Hence, comorbidities of any cause may increase vulnerability to severe TBE.

The median CSF-protein and CSF/S-albumin ratio increased with increasing disease severity. Comparingly, one previous retrospective study reported an association between high CSF-protein levels and increased risk of sequelae [42]. Nevertheless, the absolute values of CSF-protein and CSF/S-albumin-ratio were only slightly elevated, even for the severely affected patients; consequently, these variables are of limited clinical utility in assessing TBE severity. Our study showed that women had greater disease risk than men did. Moreover, severity did not increase with age, which is uncommon compared to other studies [40, 43]. We do not have any reasonable explanation for these findings based on our data.

In the present study, patients with increasing disease severity, grouped as mild, moderate or severe, had significantly increased total CCS score. This finding suggests that our modified CCS is a useful measure of TBE severity. Moreover, the CCS might be a useful tool for tracking changes over time, enabling clinicians to assess the long-term consequences of TBE.

Our study was carried out conscientiously due to the close collaboration of a few experienced physicians. The clinical characteristics and supplementary findings during hospitalization are more extensive than those from most previous studies. However, a consequence of being extensive and performing many analyses for a relatively small sample is that some of the results may be type 1 errors, i.e., false-positives. Therefore, we present Bonferroni-corrected p values. However, although Bonferroni correction is a conservative method, we cannot rule out the possibility of type II errors.

Even though we used previously defined levels of TBE severity, the classification of disease severity might be considered a methodological limitation. For example, some patients who were classified as having a mild disease reported balance disorders and/or concentration difficulties and memory changes. Although such findings may be observed with meningitis, the proportion of patients with moderate disease might have been underreported in patients in whom subtle manifestations of encephalitis were not obvious. Similarly, classifying patients was challenging when assessing the difference between “slightly altered consciousness” and “altered consciousness”. This challenge led to repeated discussions within the study group to ensure proper classification. Moreover, some symptoms fluctuated during the acute phase, possibly leading to underreporting. However, the patients were closely observed and monitored, reducing the risk of underreporting. Not all patients underwent certain investigations, such as EEG, MRI or lumbar puncture, which is a limitation.

Of the patients included three months or more after admission, more than half were investigated at follow-up visits. The majority of these patients were already known to the physician from the time of TBE hospitalization. Consequently, we consider recall bias a minor limitation in the present study.

In conclusion, this study highlights several aspects of TBE, including the high prevalence of a monophasic course, the prevalence of dysesthesia, the possibility of TBE without pleocytosis, the high proportion of patients with hyponatremia and the impact of preexisting comorbidities on disease severity. Asymptomatic comorbidities account for a substantial proportion of the comorbidities. Therefore, vaccination may be crucial for people with any underlying disease living in TBE endemic areas of TBE. Moreover, when patients in endemic areas present with dysesthesia during the tick season, clinicians should consider TBE as a differential diagnosis. However, further investigations are needed to identify which patients are susceptible to long-term sequelae and to understand the impact of TBE on quality of life. Exploring biomarkers to predict disease severity and outcome would be helpful in illuminating even more aspects of TBE.

### Electronic supplementary material

Below is the link to the electronic supplementary material.

Supplementary Material 1

## Data Availability

The datasets generated during and/or analyzed during the current study are not publicly available because the study is still ongoing; however, they are available from the corresponding author upon reasonable request. The data were handled in compliance with the General Data Protection Regulation (GDRP) requirements. The data are stored on TSD (Tjeneste for Sensitive Data) facilities owned by the University of Oslo, operated, and developed by the TSD service group at the University of Oslo, IT-Department (USIT) (tsd-drift@usit.uio.).
