# Supplementary material for: Clinical characteristics and factors affecting disease severity in hospitalized tick-borne encephalitis patients in Norway from 2018 to 2022
Source: Eur J Clin Microbiol Infect Dis. 2024 May 27;43(7):1355–66. doi: 10.1007/s10096-024-04855-2 (PMC11271349; doi:10.1007/s10096-024-04855-2)
Supplement: Supplementary file 1 — Supplementary Material 1 [file 10096_2024_4855_MOESM1_ESM.docx]

**Supplementary information**

**Modified Composite clinical score**

| **Modified Composite clinical score** |
| --- |
|  |
| *Subjective symptoms related by the patient to the current tick-borne encephalitis (TBE)* |
|  |
| Malaise |
| Fatigue |
| Headache |
| Nausea |
| Neck stiffness |
| Abdominal and/or breast pain |
| Arm pain |
| Leg pain |
| Generalized pain located to joints and/or muscles |
| Sensitivity to light |
| Sensitivity to sound |
| Anxiety, mood swing and depression |
| Memory difficulties |
| Concentration difficulties |
| Balance disturbance |
| Sleep disorders (related to currently TBE) |
| Other |
|  |
| *Peripheral findings related to the current TBE* |
|  |
| Paresis of the eye muscles. |
| Reduced hearing |
| Other cranial neuropathies |
| Cervical-, thoracic, - and/or lumbar radicular sensory findings* |
| Cervical radicular paresis† |
| Lumbar radicular paresis† |
| Nonradicular sensory findings‡ |
| Nonradicular paresis§ |
| Other |
|  |
| *Central findings related to the current TBE* |
|  |
| Neck stiffness |
| Facial palsy |
| Central findings in one extremity ¶ |
| Central findings in a hemi pattern |
| Central findings in both legs |
| Central findings in all extremities |
| Gait ataxia |
| Balance disturbance (by testing) |
| Dysphasia/aphasia |
| Nystagmus |
| Involuntary movement including tremor |
| Cognitive symptoms |
| Other |
|  |
| *Abnormal sensory pattern in a radicular pattern. |
| †Paresis in a radicular pattern. |
| ‡Sensory findings matching with a peripheral nerve or plexus. |
| §Paresis matching a peripheral nerve or plexus. |
| ¶Central weakness and/or spasticity, impairment in pace or fine motor skills |
